# Effectiveness and safety of traditional Chinese medical bath therapy combined with ultraviolet irradiation in the treatment of psoriasis: A systematic review and meta-analysis of randomized controlled trials

**DOI:** 10.1371/journal.pone.0173276

**Published:** 2017-03-21

**Authors:** Jingzhi Guan, Shaofei Yuan, Hanqimuge Wu, Risu Na, Xueqin Wu, Xin Wang, Shan Bao

**Affiliations:** 1 Pharmaceutical Department, Inner Mongolia International Mongolian Hospital, Hohhot, Inner Mongolia, China; 2 Pharmaceutical Department, The Second Affiliated Hospital of Baotou Medical College, Baotou, Inner Mongolia, China; 3 Pharmaceutical Laboratory, Inner Mongolia International Mongolian Hospital, Hohhot, Inner Mongolia, China; 4 Department of Psychosomatic Medicine, Inner Mongolia International Mongolian Hospital, Hohhot, Inner Mongolia, China; University of Alabama at Birmingham, UNITED STATES

## Abstract

**Background and objective:**

To systematically evaluate the clinical effects and safety of traditional Chinese medical bath therapy (TCMBT) combined with ultraviolet irradiation in the treatment of psoriasis.

**Methods:**

Electronic database retrieval was utilized. The foreign retrieval databases consulted included those of the Cochrane Library, PubMed and EMBASE; the domestic retrieval databases included the Chinese Biomedical Literature Database (Sino-Med), the China National Knowledge Infrastructure (CNKI), VIP and the WangFang Database. Clinical randomized controlled trials were conducted to evaluate the effects of TCMBT combined with ultraviolet irradiation in the treatment of psoriasis; the language of the retrieved articles was Chinese or English. Each database was searched from its inception to August 1, 2015. Two researchers independently collected the data and analyzed the methodology of the documented literature. The researchers conducted a meta-analysis with RevMan 5.2.3 software.

**Results:**

According to the available literature, 25 RCTs (randomized controlled trials) of low research quality were conducted. According to the meta-analysis, the total effective rate of TCMBT combined with ultraviolet irradiation was relatively higher than that of ultraviolet irradiation alone. The recurrence rate, incidence of adverse reactions and Psoriasis Area and Severity Index (PASI) for the combined therapy was lower than that of ultraviolet irradiation (*P<0*.*05*).

**Conclusion:**

For the treatment of psoriasis, the clinical effects and safety of TCMBT combined with ultraviolet irradiation are generally better than those of ultraviolet irradiation alone. However, the original literature was written in Chinese, and the quality of the studies was not high. Thus, it is difficult to confirm the clinical effects and safety of TCMBT combined with ultraviolet irradiation. It is necessary to conduct a scientific, normalized and high-quality RCT with multiple large samples and centers.

## Introduction

The skin is the first barrier of the human body, being a kind of heterogeneous organ, covers the whole body with the functions of defense, feeling[1], body temperature regulation, secretion and excretion function[2–3], etc. A variety of lesions can occur to skin and psoriasis is one of them. Psoriasis is a common, chronic skin disease that generally appears in young adults, with 1%-3% morbidity during the patient’s first visit to the dermatologist. Psoriasis features an agnogenic and typically red papule, with mild swelling of the skin surface. At the initial stage, there are only a few papules that cluster in later stages to form plaques. The skin surface dries and becomes covered with thick and glossy silver scales. Psoriasis is symmetrically distributed in the scalp and the medial aspect of the arms, legs, and body. There are clear boundaries between the tissues with lesions and normal tissues. Psoriasis is a type of chronic disease that is related to human immunity and inflammation. Around the world, 2%-3% of the population is affected[4]. Psoriasis is hard to cure and easy to relapse. Affected patients must subscribe to long-term therapy, often utilizing many medical resources and incurring large treatment costs. As reported by both the Dermatological Research Association and the American Academy of Dermatology, the direct and indirect treatment costs of psoriasis reaches up to 1.2 billion dollars and to 114 billion dollars a year, respectively[5]. At present, the drugs needed to treat psoriasis that are required by the world’s population would cost 3.3 billion dollars each year[6]. Psoriasis is a chronic relapsing inflammatory skin disease with the joint participation of a variety of immune cells. At present, the pathogenesis of psoriasis is not clear, and it is related to many factors, such as heredity, infection, immunity and so on. Including: First, genetic factor: psoriasis is a polygenic and recurrent genetic disease. Second, infection factor: It is caused by viral infection. Acidophilous inclusion body has been found within the epidermis spines nucleus. Streptococcal infection may be an important predisposing factor for the disease, because acute tonsillitis or upper respiratory infection is often occurred before the eruption of acute guttate psoriasis. Third, metabolic disorder: The decline of content in lipid, cholesterol, globulin, sugar, uric acid, potassium, and folic acid; it was also reported the increasing of polyamine and arachidonic acid within skin lesion. The lipid metabolism disorder is significantly related to psoriasis has also been proved, but the mechanism is not clear yet. Forth, immune disorder: Other than skin lesions for psoriasis, the monocyte and lymphocytes infiltration are obvious in the position of skin lesions. In particular, T lymphocyte dermis infiltration is an important pathological feature of psoriasis, suggesting that the immune system takes a place in the development and progression of psoriasis. Without specific diagnostic methods and efficient treatment, psoriasis is still a difficult and complicated disease. According to early records of traditional Chinese medicine, psoriasis was known as “Ma Pi Xuan or Gou Pi Xuan” in *Waike Zhengzong*. Although the disease name was diverse, the patterns and characteristics of psoriasis were precisely described [7–8]. According to *YiZong JinJian*, the cause of the disease was the dryness of the blood due to cold or exposure. *Waike Zhengzong* described the genesis of the disease as the dryness of the blood, which impacted both the spleen and lungs and was due to cold weather or exposure[9]. At present, it is known that psoriasis is related to blood temperature, blood dryness, blood stasis or exposure to the cold. Some experts considered that psoriasis was related to exposure to the wind, moisture, heat, dryness, which was reflected in the skin surface so that the blood circulation was out of balance with qi stagnation, blood stasis and skin malnutrition[10–11]. Western medical therapies were focused on hormonal changes, antihistamine medications, and ultraviolet irradiation, among others[12–14]. However, the curative effect and safety of these interventions was not favorable. UVB in narrow-spectrum is one of important means for clinical treatment of psoriasis but its mechanism has not been fully determined. It may include effects, such as, the release of induced apoptosis[15], cytokines that has influence on various of cytokines, immunosuppression, the regulation of immunosuppression and endocrine regulation[16–19], anti-angiogenesis and anti-proliferation, etc. to make the psoriasis skin lesion return to normal. It’s widely applied clinically due to its characteristics, such as, high curative effect, high curative effect and long catabasis. Based on the fundamental theory of traditional Chinese medicine (TCM), the therapeutic methods of traditional Chinese medicine bath therapy were widely used in the treatment of psoriasis[20–23]. TCMBT was the quintessential external application of TCM. During the TCMBT process, liquid was kept in a vessel, and a body part or the whole body was soaked. Through the physical effect of medical therapy, hydrotherapy, and thermal therapy, TCMBT was designed to treat psoriasis and maintain health through stimulation of the skin and its main and collateral channels, administration of acupuncture, and transdermal absorption of drugs. TCM differentiation was applied to understand its pathogenic factor and diseased region. Using multiple therapies, TCM differentiation could reduce the toxicity and side effects of Western medical therapies. In recent years, the medicated TCM bath in the treatment of psoriasis has been widely recognized by clinicians worldwide.

## Methods

### Ethics statement

All the analyses were based on previously published studies; thus, ethical approval and patient consent were not required.

In this study, we systematically evaluated randomized controlled trials that used TCMBT combined with ultraviolet irradiation in the treatment of psoriasis and that were completed and published before August 1, 2015. The existing literature on the treatment effectiveness and safety of TCMBT was analyzed and expected to provide a reliable theory for the treatment of psoriasis based on TCMBT and combined therapy of TCMBT with Western medicine.

### Search strategy

Computer-based retrieval technology was applied to the electronic databases. We searched seven databases. The foreign databases included the Cochrane Library, PubMed and EMBASE, and the domestic databases included SinoMed, CNKI, VIP, and the WangFang Database. The retrieval of subject terms and free text was used. For example, Chinese words such as “Yao Yu”, “Zi Wai”, and “Yin Xie Bing” and English words including “traditional Chinese medicine”, “bath”, “dipping”, “psoriasis”, “psoriases”, “palmoplantaris pustulosis”, “pustulosis palmaris et plantaris”, “pustular psoriasis of palms and soles”, “UVA”, “UVB”, and “ultraviolet” were searched. Chinese and English research containing search terms was available from the database’s date of launch up to August 1, 2015. The manual retrieval was limited to *Lishizhen Medicine And Materia Medica Research*, *the Chinese Journal of Experimental Traditional Medical Formulae*, *the Journal of Clinical Dermatology*, *The Chinese Journal of Dermatovenereology*, *the Liaoning Journal of Traditional Chinese Medicine* and other related sources that were published in 2010 or thereafter. At the same time, it was necessary to perform an additional retrieval to improve the recall ratio.

Inclusion and Exclusion Criteria

### Types of studies

Clinical randomized controlled trials describing TCMBT combined with ultraviolet irradiation in the treatment of psoriasis have been conducted; these studies were written in Chinese or English. The retrieval time was from the launch date of each database to August 1, 2015. There were no retrieval limitations based on a patient’s age, gender or the origin of their case. The diagnostic criteria for psoriasis came from 《Dermatology and Venereology》, 《Modern Dermatovenereology》, 《Clinical Dermatology》, 《Yang Guoliang Dermatology》 and 《China Clinical Dermatology》[24–28].

### Intervention measures

The patients in the experimental group were treated with a traditional Chinese medical bath combined with ultraviolet irradiation (the dosage, timing and interval, treatment course, and attributes of ultraviolet irradiation were not limited). The patients in control groups were treated with a single dose of ultraviolet irradiation (the treatment time, exposure intensity, interval of treatment and attributes of ultraviolet irradiation were not limited).

### Types of outcome measures

① The total effective rate, refers to the grading of PASI where the criteria were classified into cured, excellent and valid cases[(Incidence of Recovery: The decline of PASI integral is more than or equal to 95%; Incidence of remarkable effect: The decline of PASI integral is from 60% to 94%; Incidence of effectiveness: The decline of PASI integral is from 20% to 59%; Incidence of Invalidity: The decline of PASI integral is less than 20%); Total effective rate = (the cases of recovery + the cases of remarkable effect) / total cases * 100%)];
② the recurrence rate(The relapse of disease after six months’ treatment);
③ the incidence of adverse reactions; (Dry skin, pruritus, erythema, pain and chromatosis, etc.);
④ the Psoriasis Area and Severity Index.

### Exclusion standards

① Non-RCT literature, ② complications, ③ inconsistent data, ④ <20 references, and ⑤ incomplete case reports, reviews, or animal experiments and requests to primary investigators for original data that went unanswered.

### Data extraction

Two researchers (Wu XQ and Wu HQMG) independently read the studies as obtained in the search. As required, the studies meeting the inclusionary criteria were selected. For studies not meeting the inclusionary criteria, various reasons for exclusion are indicated in the table. Finally, the studies were included if details such as the author’s name, publication time, gender ratio, average age, average disease course, positive cases for both test and control groups, the treatment course, diagnostic codes, curative effects, and outcome indicators were included. If the results were hard to decipher, a third researcher (Bao S) provided assistance.

Assessment of the risk of bias

The bias risk assessment tool recommended by the Cochrane Review Handbook 5.2 was used, which included required evaluations of 1) random allocation, 2) allocation concealment, 3) the decision of blinding-method on research subjects, (including who implemented treatment and surveyed outcomes), 4) data integrity, 5) research results of selective reports, 6) other sources of bias (i.e., publication bias, drug compatibility bias, drug dose bias, search bias, English language bias, and multiple publication bias) etc.)[29]. Two researchers (Wu XQ and Wu HQMG) would independently make the evaluation and cross-check the evaluation results. If the results were difficult to decipher, a third researcher (Bao S) provided assistance.

### Data analysis

Rev Man 5.3 software was used to perform the meta-analysis. In the paper, OR and 95% CI were applied. In terms of identical measurements, WMD and 95% CI were introduced. Since the same variable had the different measurements, MD and 95% CI were introduced. According to the differences in the findings, X^2^ tests were introduced (α = 0.1), and based on the I^2^ variable, the differences in the findings were judged. If the research results had no significant difference (P>0.1, I^2^≤50%), the fixed-effect model based on the meta-analysis was introduced. If the research results were significantly different (P≤0.1, I^2^>50%), the random-effect model based on the meta-analysis was introduced as long as the obvious difference of the research results were excluded. If the research results were significantly different, then subgroup, sensitivity, or descriptive analysis was introduced.

## Results

### Study selection

**Fig 1:**
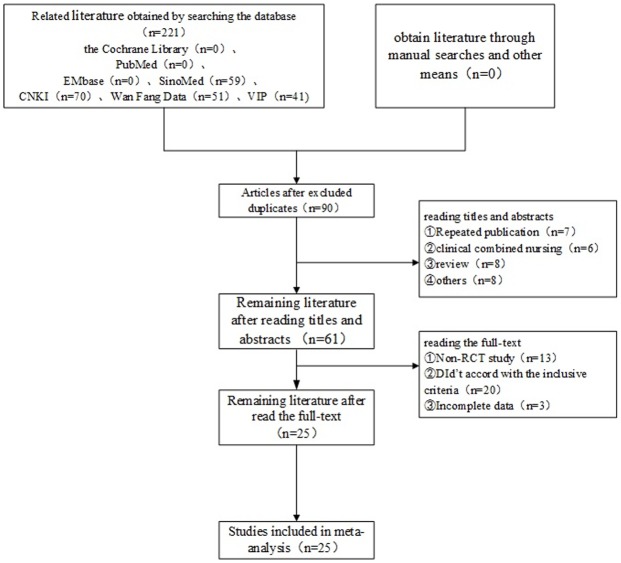
Study selection flow chart.

The database search obtained 221 studies according to the retrieval strategy and data collection methodology (all studies were Chinese in origin). The results from each database are as follows: the Cochrane Library (n = 0), PubMed (n = 0), EMBase (n = 0), SinoMed (n = 59), CNKI (n = 70), the Wan Fang Database ((n = 51), and VIP (n = 41). First, we obtained 90 articles after removing duplicate documents with the document management software NoteExpress2. Second, after reading the titles and abstracts of the obtained studies, 29 studies were excluded [repeated publication (n = 7), ② clinical combined nursing (n = 6) ③ review (n = 8)], ④ others (n = 8)]. Thus, 61 studies available for analysis. Third, after reading the full text of each study, 36 studies were excluded [① non-RCT study (n = 13), ② did not meet the inclusion criteria (n = 20) ③ incomplete data (n = 3)]. Ultimately, 25 studies were included (Fig 1).

### Research characteristics

Ultimately, 25 trials were selected based on the inclusion and exclusion criteria. The range of sample sizes was as low as 60- cases with a mid-range of 383 cases, and in some studies sample size was as large as 3,570 cases, which included 1,839 cases in the experimental group and 1,731 cases in the control group. The basic characteristics of the studies are shown in Table 1.

**Table 1:** The basic characteristics of the included studies.

Author/Year	Groups	Sample size	Age (median or mean or range)	Course of disease	Course of treatment (days)	Intervention	Type of psoriasis	Outcomes
**Zhou and Huang, 2005**[30]	EG	143	18–60	4–28 years	28	Coptis chinensis, 10 g; Phellodendron, 20 g; Sophora flavescens, 20 g; garden burnet, 20 g; mahoni, 60 g; pomegranate rind, 15 g; broom cypress fruits, 15 g; groundsel 30 g.	(1)	①
	CG	129	18–61	5–30 years	28	UV-A	(1)	
**Liu et al., 2005**[31]	EG	88	13–60	6.06 years	40	Medicated bath: 30 minutes+NB-UVB	(1)	①
	CG	78	14–58	6.22 years	40	NB-UVB	(1)	
**Liu et al., 2005**[32]	EG	40	15–69	6.8 years	40	Cynanchum paniculatum, 30 g; Fructus Kochiae, 30 g; purslane, 30 g; Cortex Dictamni, 30 g; white hellebore, 30 g.	(1)	①
	CG	40	10–65	2.0 years	40	Medicated bath: 30 minutes+NB-UVB	(1)	
**Liu et al., 2005**[32]	EG	72	12–62	7.36 years	40	NB-UVB	(1)	①
	CG	65	13–69	7.05 years	40	Cynanchum paniculatum, 30 g; Fructus cnidii, 30 g; Sophora flavescens, 30 g; Zanthoxylum, 30 g; Patrinia, 30 g; Polygonum cuspidate, 30 g; purslane, 30 g; salvia, 20 g; Atractylodes, 15 g.	(1)	
**Liu et al., 2008**[34]	EG	89	13–60	6.72 years	28	Medicated bath: 30 minutes+NB-UVB	(1)	①②③
	CG	96	14–58	7.23 years	28	NB-UVB	(1)	
**Cui et al., 2008**[35]	EG	62	45.82±13.89	5.77±4.95 months	56	Medical stone. Medicated bath: 20 minutes+NB-UVB	(1)	①③④
	CG	57	38.72±12.17	4.47±3.76 months	56	NB-UVB	(1)	
**Sun et al., 2010**[36]	EG	35	18–60	6.72 years	56	Salvia, 30 g; Angelica sinensis, 30 g; selfheal, l30 g; Fructus Kochiae, 30 g; Cortex Dictamni, 30 g; golden cypress, 30 g; Folium Isatidis, 30 g; Smilax glabra, 30 g.	(1)	①③④
	CG	33	19–58	7.23 years	56	Medicated bath: 30 minutes+NB-UVB	(1)	
**Zhang et al., 2010**[37]	EG	30	15–60	10.37±9.59 days	56	NB-UVB	(1)	①④
	CG	30	12–58	10.85±10.46 days	56	Galla chinensis, golden cypress, Angelica sinensis, turmeric, Fructus Psoraleae.	(1)	
**Luo et al., 2010**[38]	EG	88	--	--	60	Medicated bath: 20 minutes+NB-UVB	(1)	①②③
	CG	85	--	--	60	NB-UVB	(1)	
**Lin et al., 2010**[39]	EG	95	29.52±6.38	8.25±3.16 year	48	Golden cypress, 30 g; purslane, 30 g; salvia, 30 g; Cortex Dictamni, 30 g; Angelica sinensis, 30 g. Medicated bath: 30 minutes+NB-UVB	(1)	①③④
	CG	90	27.42±6.28	7.48±2.86 years	48	NB-UVB	(1)	
**Shi et al., 2011**[40]	EG	170	7–70	12.9 years	56	Sophora flavescens, 100 g; Fructus cnidii, 50 g; Rehmannia, 50 g; Chrysanthemum indicum, 100 g; Rhizoma Curcumae, 50 g; mirabilite, 200 g; Herba Speranskiae Tuberculatae, 100 g; Chinese mugwort leaf, 100 g. Medicated bath: 20 minutes+NB-UVB	(1)	①②③
	CG	168	8–65	13.5 years	56	NB-UVB	(1)	
**Song et al., 2011**[41]	EG	193	48.2±11.5	6.52 years	28	Salvia, safflower, Rehmannia, Folium Isatidis, Cortex Moutan, Smilax glabra, red peony root, Angelica sinensis, Sparganium stoloniferum, Rhizoma Curcumae, Caulis Spatholobi, Capsicum annuum, dried alum.	(1)	①②④
	CG	190	50.2±8.6	7.23 years	28	Medicated bath: 30 minutes+NB-UVB	(1)	
**Qu et al., 2011**[42]	EG	95	36±12.4	5.7±3.2 years	40	NB-UVB	(1)	①③④
	CG	95	35±12.7	6.2±3.5 years	40	Smilax glabra, 30 g; Fructus Kochiae, 15 g; Angelica sinensis, 20 g; Cortex Dictamni, 20 g; Cynanchum paniculatum, 20 g; Sophora flavescens, 15 g; Fructus cnidii, 20 g; cocklebur fruit, 15 g; Radix stemonae, 15 g; Cortex Pseudolaricis, 10 g; Tribulus terrestris, 15 g; rhubarb, 10 g.	(1)	
**Wang et al., 2011**[43]	EG	50	18–65	8.6 years	28	Medicated bath: 20–30 minutes+NB-UVB	(1)	①③
	CG	50	19–60	7.8 years	28	NB-UVB	(1)	
**Wang et al., 2012**[44]	EG	30	16–68	1 month-25 years	30	Speranskiae Tuberculatae, Cacumen Biotae, Cortex Dictamni, salvia, Angelica sinensis, peach seed, Sophora flavescens, Fructus Kochiae, pearl powder. Medicated bath: 20 minutes+NB-UVB	(1)	①③④
	CG	30	21–70	2 months-20 years	30	NB-UVB	(1)	
**Re et al., 2012**[45]	EG	42	19–58	--	40	Gentiana macrophylla, 30 g; Acorus tatarinowii, 30 g; Sophora flavescens, 30 g; Dioscorea nipponica Makino, 30 g; paniculate swallowwort root, 30 g; Cacumen Biotae, 30 g; paper mulberry fruit leaves, 30 g; figwort root, 30 g. Medicated bath: 30 minutes+	(1)	①
	CG	40	19–58	--	40	NB-UVB	(1)	
**Han et al., 2013**[46]	EG	75	17–59	35 days-34 years	40	NB-UVB	(1)	①③④
	CG	75	18–58	28 days-35 years	40	pine tar (10%). Medicated bath: 20 minutes+NB-UVB	(1)	
**Wang, 2013**[47]	EG	112	39.75±10.69	5.46±1.24 years	20	NB-UVB	(1)	①③④
	CG	56	38.98±11.23	5.69±1.29 years	20	Radix scutellariae, Patrinia, dandelion, red peony root, Paeonia suffruticosa, salvia, Radix asparagi, Radix rehmaniae recens, Cortex Dictamni, Fuctus Kochiae, Atractylodes.	(1)	
**Wang et al., 2013**[48]	EG	60	--	--	56	Medicated bath: 30 minutes+	(1)	①
	CG	63	--	--	56	NB-UVB	(1)	
**Li, 2014**[49]	EG	32	46.45±13.43	5.46±4.67 months	56	NB-UVB	(1)	①④
	CG	32	46.45±13.43	5.46±4.67 months	56	the bark of ash, 100 g. Medicated bath: 20–30 minutes+	(1)	
**Wang and Gao, 2014**[50]	EG	53	--	--	45	NB-UVB	(1)	①④
	CG	50	--	--	45	NB-UVB	(1)	
**Luo et al., 2014**[51]	EG	56	--	--	24	Angelica sinensis, 30 g; salvia, 30 g; Fructus Kochiae, 30 g; selfheal, 30 g; golden cypress, 30 g; Cortex Dictamni, 30 g; Smilax glabra, 30 g; Folium isatidis, 30 g.	(1)	①②③
	CG	52	--		24	Medicated bath: 30 minutes+NB-UVB	(1)	
**Zhou and Yin, 2014**[52]	EG	42	46.2±2.3		56	NB-UVB	(1)	①③
	CG	42	46.2±2.3		56	Sophora flavescens, Chrysanthemum indicum, Cynanchum paniculatum, Viola philippica, Fructus cnidii, honeysuckle, Radix glycyrrhizae.	(1)	
**Zhang, 2014**[53]	EG	57	35.2±6.7		--	Medicated bath: 15–20 minutes+	(1)	①④
	CG	55	37.1±6.2		--	NB-UVB	(1)	
**Zhao et al., 2015**[54]	EG	30	21.64±8.9		--	NB-UVB	(1)	①
	CG	30	23.64±6.9		--	pine tar (10%). Medicated bath: 20 minutes+NB-UVB	(1)	

(1) psoriasis vulgaris;

① total effective rate; ② recurrence rate; ③ incidence of adverse reactions; and ④ psoriasis area and severity index.

EG: experimental group, CG: control group.

### A summary of the risk of quality and bias in the literature

**Fig 2:**
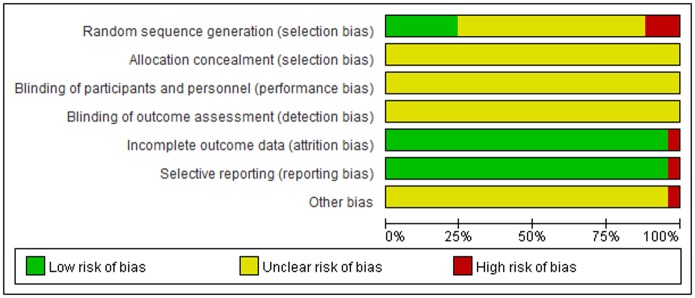
Risk of bias summary and graph.

**Fig 3:**
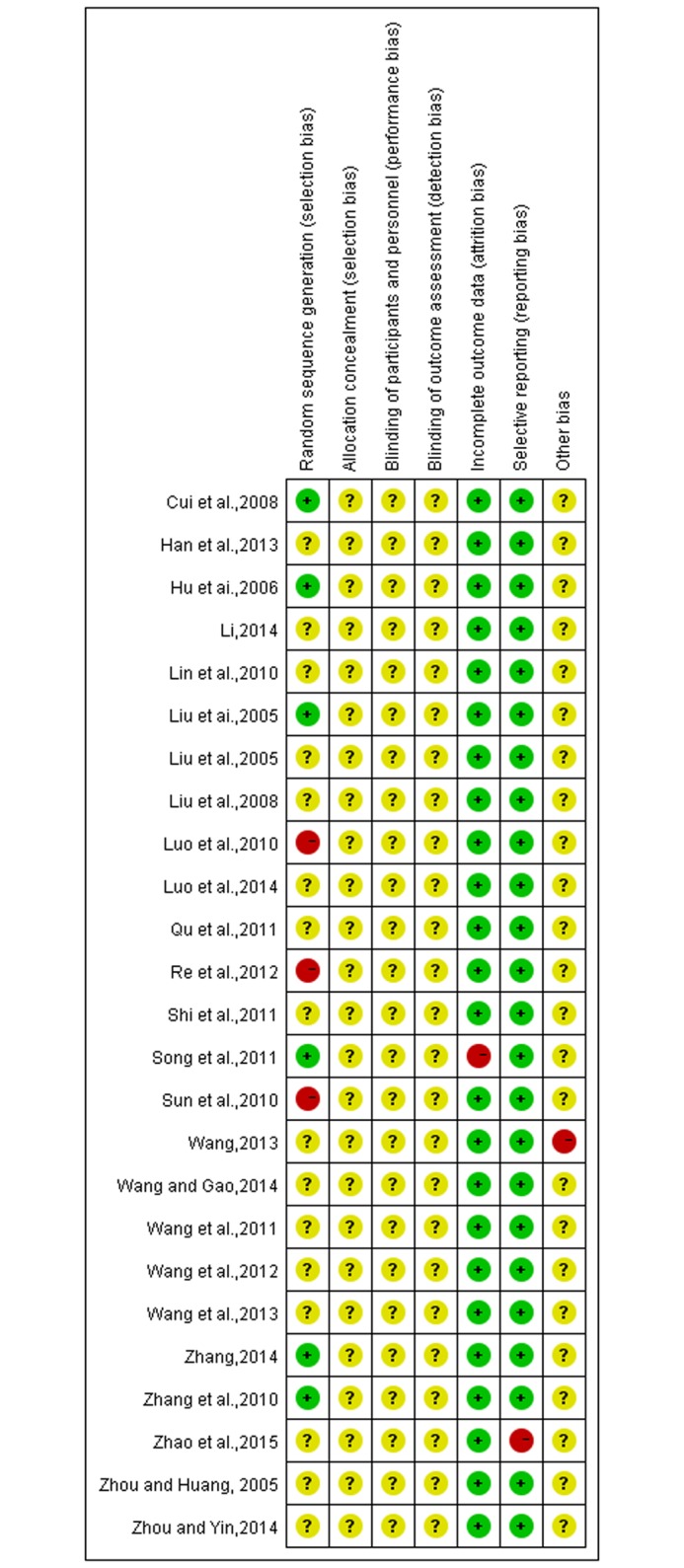
Risk of bias summary and graph.

The quality of the included trials was relatively low. All trials were described as randomized controlled trials, but only six studies reported a specific method of randomized allocation; the others only mentioned randomization[31,33,35,37,41,43]. The results of risk of bias are shown in Figs 2 and 3.

### Outcome measures

Efficacy-rate was reported in 25 studies [30–54] while 5 studies reported recurrence rate [34,38,40–41,51]. Adverse reactions were reported in 13 studies[34–36,38–40,42–44,46–47,51]and 12 studies reported using the Psoriasis Area and Severity Index[35–37,39,41–42,44,46–47,49–50,53].

### Division of subgroups

Cases in the literature were divided into two groups, according to whether the sample size of the experimental group and the control group was consistent: (1) sample size of the experimental group = sample size of the control group, and; (2) sample size of the experimental group ≠ sample size of the control group.

### Total effective rate

**Fig 4:**
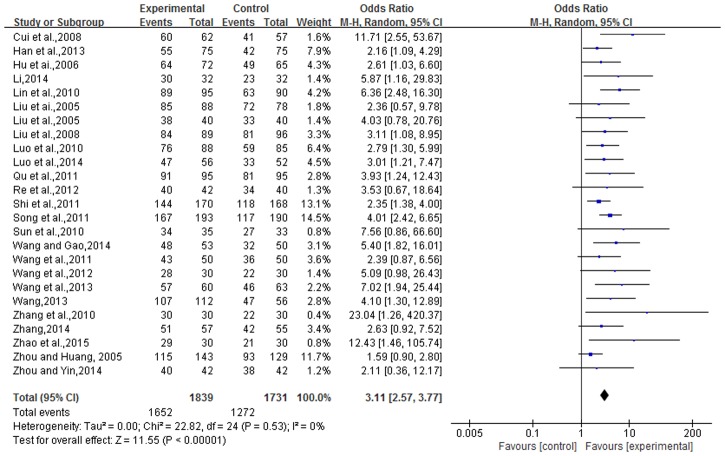
Comparison of the total effective rate of TCMBT plus ultraviolet irradiation treatment compared to ultraviolet irradiation treatment.

Efficacy-rate was reported in 25 studies, which equated to 3,570 cases. The experimental group contained 1,839 cases with, 1,731 cases in the control group. There was no heterogeneity in statistical analysis (P = 0.53, I^2^ = 0%), so a fixed-effect model was performed. The result of the meta-analysis showed that the total effective-rate in the TCMBT combined with ultraviolet irradiation treatment group was higher than the rate of those patients in the control group, and there was a statistically significant difference between the two groups [n = 25, OR = 3.25, 95% CI (2.69, 3.93), Z = 12.24, P<0.00001], (Fig 4).

### Recurrence rate

**Fig 5:**
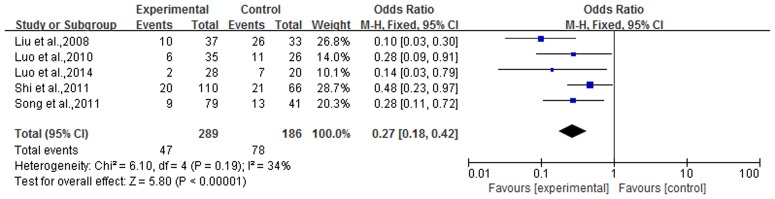
Comparison of recurrence rate between TCMBT plus ultraviolet irradiation versus ultraviolet irradiation treatment.

Recurrence rate was reported in 5 articles, for a total of 475 cases. The experimental group contained 289 cases with, 186 cases in the control group. There was no heterogeneity in the statistical analysis (P = 0.19, I^2^ = 34%), so a fixed-effect model was performed. The result of the meta-analysis showed that the recurrence rate in TCMBT combined with ultraviolet irradiation treatment groups had lower rates than those in the control group and there was a statistically significant difference between the two groups [n = 5, OR = 0.27, 95% CI (0.18,0.42), Z = 5.80, P<0.00001] (Fig 5).

### Incidence of adverse reactions

**Fig 6:**
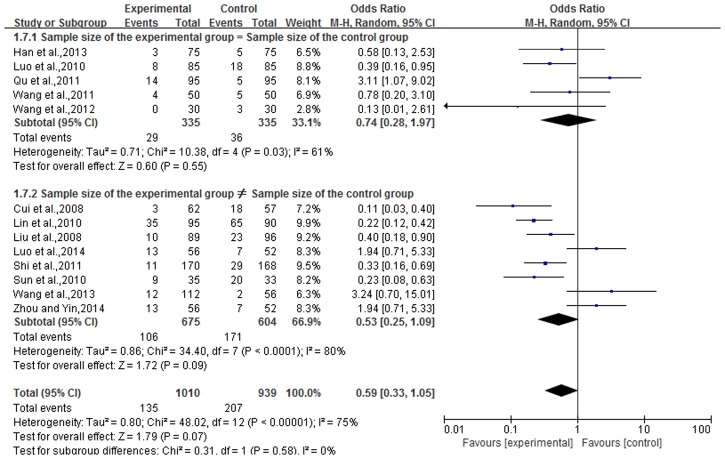
The incidence of adverse reactions in TCMBT plus ultraviolet irradiation compared to ultraviolet irradiation treatment.

Adverse reactions were reported in 13 articles reported, for a total of 1,969 cases. Experimental groups contained 1,030 cases and there were, 939 cases in the control group. There was heterogeneity in the statistical analysis (P<0.01, I^2^ = 75%), so an analysis was performed by dividing subgroups and using a random-effects model. The results of subgroups (sample size of the experimental group = sample size of the control group and sample size of the experimental group ≠ sample size of the control group) showed that there were not a statistically significant difference between the two groups [subgroup 1, n = 5, OR = 0.74, 95% CI (0.28,1.97), Z = 0.60, P = 0.55; subgroup 2, n = 8, OR = 0.53, 95% CI (0.25,1.09), Z = 1.72, P = 0.09]. The overall effect showed that the incidence of adverse reactions in TCMBT combined with ultraviolet irradiation treatment groups was lower than the rate of those in the control group and there was no statistically significant difference between the two groups [n = 13, OR = 0.59, 95% CI (0.33,1.05), Z = 1.79, P = 0.07>0.05] (Fig 6).

### Psoriasis area and severity index

**Fig 7:**
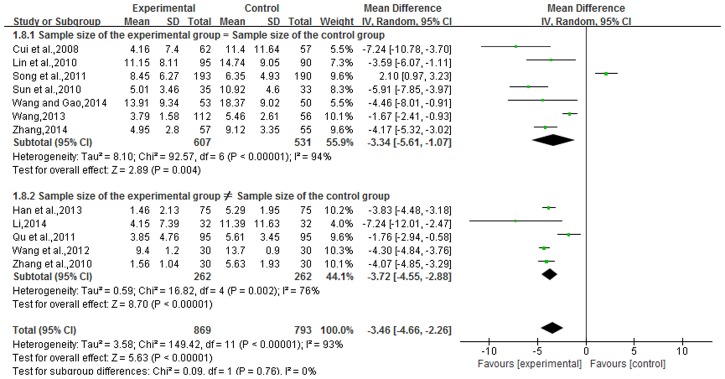
The incidence of adverse reactions in TCMBT plus ultraviolet irradiation compared to ultraviolet irradiation treatment.

The Psoriasis Area and Severity Index was reported in 12 articles, for a total of 1,662 cases. Experimental groups contained 869 cases, with 793 cases in the control group. There was heterogeneity in the statistical analysis (P<0.01, I^2^ = 93%), so an analysis was performed by dividing subgroups and using a random-effects model., The results of subgroups (sample size of the experimental group = sample size of the control group and sample size of the experimental group ≠ sample size of the control group) showed that there was a statistically significant difference between the two groups [subgroup 1, n = 7, OR = -3.34, 95% CI (-5.61, 1.07), Z = 2.89, P = 0.004; subgroup 2, n = 5, OR = -3.72, 95% CI (-4.55, 2.88), Z = 8.70, P<0.00001]. The overall effects showed that the Psoriasis Area and Severity Index in TCMBT combined with ultraviolet irradiation treatment groups had lower scores than those patients in the control group and there was a statistically significant difference between the two groups [n = 12, MD = -3.46, 95% CI (-4.66, 2.26), Z = 5.63, P<0.00001] (Fig 7).

### Publication bias

**Fig 8:**
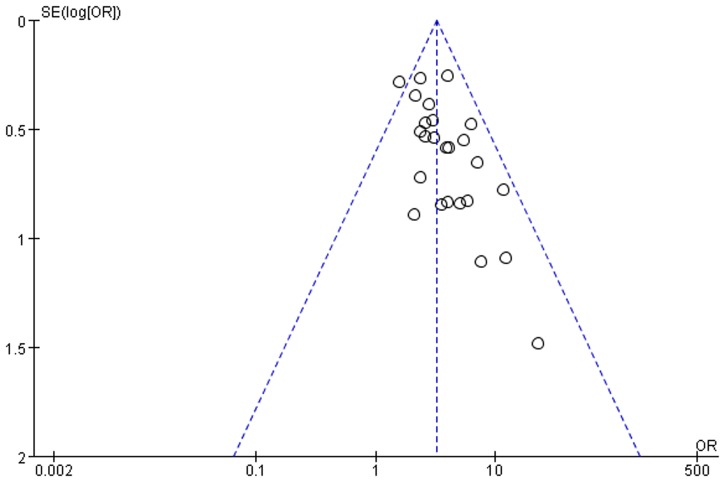
Funnel plot total effective rate of TCMB + ultraviolet irradiation treatment compared to ultraviolet irradiation treatment.

In terms of those 25 studies, the value of the OR of each study was set as the horizontal plain while the standard error-logOR value was set as the vertical plain. This system was designed to draw the funnel plot of the total effective rate of TCMBT with irradiation therapy. According to the analysis results, it was found that there was no symmetric distribution on either side of the plot. These results indicate that the published literature might contain some publication bias and might be related to fewer publications regarding the negative results (Fig 8).

## Discussion

TCMBT is both an important part of external therapy and a branch of hydrotherapy in traditional Chinese medicine. Based on the fundamental theory of traditional Chinese medicine, the partial or the whole-body parts were dipped into the liquid. Through the medication properties and the water attributes, TCMBT was designed to cure the disease. TCMBT was featured as a rapidly functioning, easily learned, operated, and promotable, safe treatment with, lower toxicity and fewer side effects with an extensive application. It was particularly suitable for the young, the old, and individuals who found daily medication regimens difficult or who, were unwilling to or could not take their medication. TCMBT has the same advantages with oral administration and would achieve the satisfactory effect for a patient with incurable skin disease. The external therapy of a medicated bath has an impact on the internal treatment of integumentary disease. The holistic concept of traditional Chinese medicine considers that the human body is an organic sum of its parts and that each part of the human body is indivisible. It is mutually coordinating and interactional. Although the external therapy is different from the internal therapy, the channels are interactive and contact with the external tissues, organs, and viscera, harmonize qi and blood, balance the yin and yang of viscera to achieve the self-healing effect and improve the ability of disease resistance. In recent 20 years, the unique advantages in the treatment of psoriasis by NB-UVB irradiation have been fully demonstrated. With the wavelength of 311nm and strong penetrability, it can penetrate into the deep layer of skin, induce apoptosis of T lymphocytes, and inhibit the DNA synthesis of epidermis cell so as to inhibit the hyperplasia of epidermal cell. Compared with the therapies of PUVA and BB-UVB, it has the advantages of good curative effect, lower light carcinogenicity with long-term treatment, non-medication consumption requirement before treatment, safely usage by pregnant women, etc. Compared with the traditional treatment and biological systemic treatment, the adverse reaction was little[55]. At the same time, it can be shown from the research of UVB spectrum that in the course of treatment of psoriasis[56], the UVB with the wavelength of 290 ~ 300 nm can only generate erythema effect and keratinocyte necrosis, having little effect on lymphocyte infiltrated on skin lesion while the effect of wave length with the length of 311 nm is the most obvious. The ultraviolet ray with this wave length has been the mostly applied in recent years. It has good unicity, and slight erythema reaction. Therefore, 311nmNB-UVB irradiation has been considered to be an ideal method for the treatment of psoriasis in recent years. At the same time, NB-UVB also plays a certain role in terms of changing the skin secretion function [56–58].

At present, NB-UVB is often combined with western medicine in treatment of psoriasis because it can reduce the usage amount of drugs and the total dosage of phototherapy, having the advantages of quick effect, high curative effect, and less time-consuming, etc. But the adverse reactions generated in the process of phototherapy cannot be ignored. In China, NB-UVB is often combined with medicated bath of traditional Chinese medicine in the treatment of psoriasis. After the skin soaks and baths in medicated bath, the effusion and pollutant will be removed; besides, it plays an effect on skin moisture and itching, heat clearing& blood cooling, and detoxification & convergence. It can expand the dilate capillaries, improve local microcirculation, promote metabolism, and accelerate tissue repair; The combination of NB-UVB irradiation in the treatment of psoriasis is beneficial to the ultraviolet penetration, enhances the radiation response of NB-UVB, and gradually improves the pathological changes, thus accelerating the repair of skin lesions. Besides, some components in traditional Chinese medicine have protective effect on radiation injury so as to reduce the adverse drug reaction incidence of phototherapy[59].

In this paper, we conducted a systematic review regarding the clinical efficacy of TCMBT combined with ultraviolet irradiation in the treatment of psoriasis. The purpose of this article was to provide elucidation of the clinical treatment of psoriasis. In this study, 25 articles were selected and clinical efficacy was reviewed from multiple aspects, including the total effective rate, recurrence rate, the incidence of adverse reactions and the Psoriasis Area and Severity Index. The results of the analysis were concluded as follows:

First, the total effective rate and recurrence rate based on TCMBT combined with ultraviolet irradiation was higher than that of ultraviolet therapy alone, while the recurrence rate and PASI-score of the combined therapy was lower than that of a single therapy.

Second, the incidence of adverse reactions of the two methods was not significantly different and the major adverse reactions included localized issues of dry skin, desquamation, erythema, pruritus, or scorching hot and stabbing pain. There were no recorded systemic adverse reactions that occurred. It was suggested that the results might be correlated with the large dose of medications and the longer exposures to the medicated bath and ultraviolet irradiation. The incidence of adverse reactions might be decreased as long as the medication dosage and the medicated bath and ultraviolet irradiation exposure was properly adjusted.

The quality evaluation of selected articles adopted the bias risk assessment tool recommended by the Cochrane Review Handbook 5.2. This study selected a total of 25 articles, but all were low quality articles. The correct grouping method (random number table method and random lottery method) were mentioned and adopted in five articles[31,33,35,37,53] with the result of "low risk of bias" obtained. 3 articles[36,38,45] adopted the incorrect block method (patients were divided according to the requirements of the patients) with the result of "high risk of bias" obtained. The rest articles were mainly mentioned and randomly divided, so we can not know whether the grouping method is correct or not with the result of "Unclear risk of bias” to give results obtained; All the selected articles failed to provide enough information, so we can not determine whether the selected articles exists or not. The given result was “Unclear risk of bias” because of allocation concealment and the blinding of the outcome assessment. One article[41] reported the case of loss to follow-up, and the rest of the articles all did not mention data integrity with the result of “low risk of bias” thereby obtained therefore; One article[54] had research results of selective reports and the rest of the literatures all didn’t show up the existed research results of selective reports; Although the quality of the selected articles are relatively lower, having a certain impact on the results of the study, but the study results are relatively stable with subgroup analysis adopted regarding the results with large heterogeneity so that the obtained conclusion from the research is reliable.

## Limitations and advantages

The advantages of this study are as follows: First, only the UV-light was adopted regarding the treatment method in the control group without any drug therapy applied so that it can highlight the therapeutic effect of medicated bath in the treatment group. At the same time, the applied PASI score was used as one of the evaluation indexes. The meta-analysis showed that the PASI score was not highly heterogeneous. Second, psoriasis is usually treated with oral drugs. Due to the differences in drug absorption and distribution, only a small amount of the drugs can reach the focus so that the treatment effect is reduced while the traditional Chinese medicine bath can carry out systemic immersion with the various parts of the body treated at the same time. Meanwhile, the drug can directly act on the focus so that the drug loss can be reduced and the effective drug treatment concentration can be maintained. Third, the retrieval adopts the minimum limit so as to ensure as many as possible related articles will be included in the research.

The limitations of this study are as follows: First, there are some difficulties in the interpretation of the results due to the selected research does not to adopt a unified international standard, Second, there is no comparison of traditional Chinese medicine bath with the effectiveness and safety study of placebo so that it has an impact on the result of meta-analysis, reducing the result reliability. Third, the shortage of follow-up in the partial selected studies results in unclear long-term effect and safety regarding the traditional Chinese medicine bath therapy. Forth, all of the selected articles were located in China, and there could be a potential concern of location bias; Fifth, since only Chinese subjects and Chinese or English studies were included, there could be a potential for both selection bias and English language bias[60–61]; Sixth, the different compatibility and dosage of medications might potentiate both drug compatibility bias and medication-dosing bias. Seventh, the exposure time to both the medicated bath and ultraviolet irradiation was not unified. Therefore, the research results could also be influenced[62]. However, similar research on the clinical effect was never reported[63–64]. On this basis, a new research index and research studies were added with the goal of enriching this research method.

This meta-analysis shows that, more samples would improve the statistical effect. The multiple independent studies based on the meta-analysis could improve the test effect. According to the results of the meta-analysis, the total effective rate of TCMBT combined with ultraviolet therapy in the treatment of psoriasis was presented. In addition, this combined therapy could reduce the patient’s recurrence rate and the incidence of adverse events. During this process, the literature was not comprehensive. Most of the authors did not provide a clear description of either the treatment procedure or allocation concealment and the method of blinding was not proposed. The follow-up time for visits was short and the diagnosis and judgement criteria were not consistent. Therefore, the credibility of the evaluation could be influenced.

## Conclusion

As previously mentioned, powerful and robust clinical tests should be explored to evaluate the effectiveness and safety of TCMBT combined with ultraviolet irradiation in the treatment of psoriasis. In the future, it may be necessary to conduct randomized controlled trials with larger sample sizes, in multiple centers, that are of high-quality and for which important cases are followed-up with longer visits.

## Supporting information

S1 File PRISMA checklist. (DOC)

S2 File Search terms. (DOCX)

## Abbreviations

CG: control group
CNKI: the China National Knowledge Infrastructure
EG: experimental group
NB-UVB: narrow bound ultra violet B light
OR: odds ratio
PASI: Psoriasis Area and Severity Index
RCTs: randomized controlled trials
Sino-Med: the Chinese Biomedical Literature Database
TCMB: traditional Chinese medical bath
TCMBT: traditional Chinese medical bath therapy
VIP: the Chinese Scientific Journal Database
WMD: weighted mean difference
