# Supplementary material for: Effectiveness and safety of traditional Chinese medical bath therapy combined with ultraviolet irradiation in the treatment of psoriasis: A systematic review and meta-analysis of randomized controlled trials
Source: PLoS One. 2017 Mar 21;12(3):e0173276. doi: 10.1371/journal.pone.0173276 (PMC5360218; doi:10.1371/journal.pone.0173276)
Supplement: S2 File — (DOCX) [file pone.0173276.s002.docx]

**Search Terms**

*CNKI(Search in All Fields). Consider the following:*

Search type A：thesaurus = Ziwai and thesaurus %= Yaoyu and thesaurus %= Yinxiebing (fuzzy matching)

*VIP (Search in All Fields). Consider the following:*

Search type: keywords or title= Yaoyu and keywords or title= Yinxiebing and keywords or title= Ziwai

*WangFang Date (Search in All Fields). Consider the following:*

Search type: thesaurus: (Yaoyu)* thesaurus: (Ziwai)* thesaurus: (Yinxiebing)* Date:-2015

*SinoMed (Search in All Fields). Consider the following:*

#6 (#5) AND (#4) AND (#3)

#5 (#2) OR (#1)

#4 "Ziwai"[All fields]

#3 "Yaoyu"[All fields]

#2 ("Yinxiebing"[All fields] OR "Nongbaobing"[All fields] OR "psoriasis "[**MeSH**])

#1 "Yinxiebing"[ unweighted: extension]

*Pubmed (Search in All Fields). Consider the following:*

#7 Search (((("Psoriasis"[Mesh]) OR #2)) AND ((dipping[Title/Abstract]) AND bath[Title/Abstract])) AND Ultraviolet[Title/Abstract] Schema: all

#6 Search (((("Psoriasis"[Mesh]) OR #2)) AND ((dipping[Title/Abstract]) AND bath[Title/Abstract])) AND Ultraviolet[Title/Abstract]

#5 Search ("Psoriasis"[Mesh]) OR #2

#4 Search (dipping[Title/Abstract]) AND bath[Title/Abstract]

#3 Search Ultraviolet[Title/Abstract]

#2 Search ((Psoriasis[Title/Abstract]) AND (Psoriases[Title/Abstract]) AND (Palmoplantaris Pustulosis[Title/Abstract]) AND Pustulosis Palmaris et Plantaris[Title/Abstract]) AND (Pustular Psoriasis of Palms[Title/Abstract] AND Soles[Title/Abstract]) Schema: all

#1 Search "Psoriasis"[Mesh]

*Cochrane Library (Search in All Fields). Consider the following:*

#5 #1 and #2 and #3 and #4

#4 traditional Chinese medicine:ti,ab,kw (Word variations have been searched)

#3 dipping:ti,ab,kw or bath:ti,ab,kw (Word variations have been searched)

#2 ultraviolet:ti,ab,kw (Word variations have been searched)

#1 Psoriasis:ti,ab,kw or Psoriases:ti,ab,kw or Palmoplantaris Pustulosis:ti,ab,kw or Pustulosis Palmaris et Plantaris:ti,ab,kw or Pustular Psoriasis of Palms and Soles:ti,ab,kw (Word variations have been searched)

*EMBASE (Search in All Fields). Consider the following:*

#11 #5 AND #6 AND #9 AND #10

#10 traditional AND chinese AND medicine

#9 #7 OR #8

#8 bath

#7 dipping

#6 ultraviolet

#5 #1 OR #2 OR #3 OR #4

#4 pustular AND psoriasis AND of AND palms AND soles

#3 pustulosis AND palmaris AND et AND plantaris

#2 palmoplantaris AND ('pustulosis'/exp OR pustulosis)

#1 'psoriasis'/exp OR psoriasis OR psoriases
